# The Human Health Impacts of the Red Imported Fire Ant in the Western Pacific Region Context: A Narrative Review

**DOI:** 10.3390/tropicalmed9040069

**Published:** 2024-03-26

**Authors:** Diego J. Lopez, Kenneth D. Winkel, Troy Wanandy, Sheryl van Nunen, Kirsten P. Perrett, Adrian J. Lowe

**Affiliations:** 1 Allergy and Lung Health Unit, The University of Melbourne, Melbourne, VIC 3053, Australia; diego.lopezperalta@unimelb.edu.au; 2 National Allergy Centre of Excellence (NACE), Parkville, VIC 3052, Australia; 3 Centre for Health Policy, Melbourne School of Population and Global Health, The University of Melbourne, Melbourne, VIC 3053, Australia; 4 Department of Clinical Immunology and Allergy, Incorporating the Jack Jumper Allergy Program, Royal Hobart Hospital, Hobart, TAS 7000, Australia; 5 College of Health and Medicine, University of Tasmania, Hobart, TAS 7000, Australia; 6 Northern Clinical School, Faculty of Medicine and Health, The University of Sydney, Sydney, NSW 2050, Australia; 7 Faculty of Medicine and Health Sciences, Macquarie University, Sydney, NSW 2109, Australia; 8 Northern Beaches Hospital, Sydney, Sydney, NSW 2086, Australia; 9 Population Allergy Group, Murdoch Children’s Research Institute, Parkville, VIC 3052, Australia; 10 Department of Paediatrics, The University of Melbourne, Melbourne, VIC 3052, Australia; 11 Department of Allergy and Immunology, Royal Children’s Hospital Melbourne, Melbourne, VIC 3052, Australia

**Keywords:** *Solenopsis invicta*, fire ant hypersensitivity, immunoglobulin E, anaphylaxis, One Health

## Abstract

**Background:** The red imported fire ant (RIFA) is one of the world’s most destructive invasive species. RIFA stings are painful and can lead to allergic reactions, including life-threatening anaphylaxis, yet health impacts remain inadequately defined. **Methods:** We searched MEDLINE (Ovid) and Google Scholar (grey literature) from inception until 20 September 2023 for articles in English using search terms related to red imported fire ants and allergies, including anaphylaxis. **Results:** Approximately a third of the population in RIFA-infested areas are stung each year. The most frequent reaction is a sterile 1–2 mm pseudo pustule on the skin. Approximately 20% of stings cause a large local reaction and between about 0.5% and 2% stings cause a systemic allergic reaction which can range from skin symptoms to life-threatening anaphylaxis. Local biodiversity is also significantly disrupted by invading RIFA and may lead to complex adverse effects on human health, from agriculture losses to expanded ranges for pathogen vectors. **Conclusions:** The potential for red imported fire ants to establish themselves as an invasive species in the Western Pacific presents a substantial and costly health issue. Successful eradication and surveillance programs, to identify and eradicate new incursions, would avoid substantial health impacts and costs.

## 1. Introduction

An invasive alien species is one that is transported by human activities outside its native range, where it becomes established and proliferates and causes adverse effects on local species and ecosystems [1]. Such species are, according to the Intergovernmental Science-Policy Platform on Biodiversity and Ecosystem Services (IPBES), one of the top five drivers of global biodiversity losses, thereby constituting a “major and growing threat to Nature and Nature’s Contribution to People” [1]. One of the most serious invasive alien species is *Solenopsis invicta Buren*, also known as the red imported fire ant (RIFA) [1]. This small (from 2 to 6 mm) ant, coppery brown in colour with a darker abdomen, is native to South America [2]. Impacting human health, local biodiversity and ecosystems, and agriculture, RIFA are ranked amongst the worst invasive alien species worldwide [1]. As illustrated by the One Health concept [3], their impacts on the ecosystem could be translated to adverse effects on human health as there is an interdependence between human, animal, and ecosystem health.

**Figure 1:**
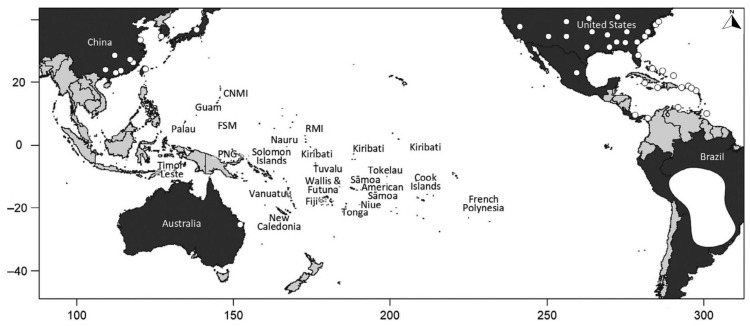
Western Pacific Region occurrence of red imported fire ants in 2021. Red imported fire ants are present in the landmasses or countries shaded in dark grey, with white points indicating regions where the ant has been recorded in the introduced range, where it is widespread in some cases (e.g., the southern United States) and localised in others (e.g., Queensland, Australia). The white mass represents the native range in South America (Gruber et al. 2021) [4].

This globally important species has, since 1930, invaded 14 states within the United States of America (USA), the Caribbean islands and, mostly from the USA [2], has travelled onwards to the Western Pacific Region (Australia, New Zealand, Taiwan, Japan, South Korea, Macau, Hong Kong and China), as shown in Figure 1 [4]. The Western Pacific Region faces heightened vulnerability to the ecological and socio-economic repercussions of invasive species. Clearly RIFA are opportunistic travellers, exploiting global shipping and various transportation modes [5], thereby posing significant threats to the small island states of the Western Pacific that receive cargo from infested areas (notably the USA).

Unlike native ant species in the region, RIFA are typically found in colonies numbering up to 250,000 ants or more, with up to 600 RIFA colonies per acre having been reported [6]. Consequently, RIFA impact the biodiversity of invaded areas, threatening diverse animal species as well as whole ecosystems, due to their aggressive nature [7]. RIFA are often found near human activity and communities [8,9], which increases the likelihood of human encounters and stinging events. Distinctively, an individual RIFA stings an average of three times before removal [9]. The stings are very painful and give the impression of being burned by fire, hence the common name [10]. Very young children or very old adults are more at risk of multiple stings as they are less mobile and therefore unable to escape effectively [9,11]. Of those stung by RIFA, around 1% can develop severe, and even life-threatening, allergic reactions [12].

The spread of RIFA and their associated impacts are expected to intensify in the near future if no preventive measures are taken [1]. Previous research determined that the eventual expansion of RIFA into the unaffected developing Pacific Island Countries and Territories could lead to significant economic costs related to socio-economical and biodiversity factors [4]. However, the RIFA-related health impacts reported in previous reviews are based on research done in the USA and the evidence dates back for many decades. Moreover, these estimates have been reported with considerable variability in the literature. A consolidated understanding of the potential human health impacts of these ants is needed to inform the decision-making process in preparedness measures and related infrastructure needs within the Western Pacific Region nations and territories. The purpose of this narrative review is therefore to summarise the current international evidence for the RIFA-related human health impacts, particularly regarding the risk of allergic reactions. As a regional health case study, we then used this evidence to predict the probable human health impacts of RIFA if they were to become established in Australia.

## 2. Materials and Methods

We undertook a comprehensive literature review to assess the potential health consequences associated with RIFA. Our investigation involved a detailed search of existing research evidence, international government reports and qualitative data.

### 2.1. Search Strategy and Selection Criteria

We searched MEDLINE (Ovid) and Google Scholar (grey literature) from inception until 20 September 2023, for articles in English using search terms related to RIFA (*Solenopsis invicta* Buren) and allergies, including anaphylaxis. The search terms used were red ant* OR fire ant* OR red fire ant* OR red imported fire ant* OR Solenopsis OR Solenopsis invicta AND allerg* OR anaphyla* AND hypersensitiv* OR sensiti* OR reaction. Additional articles were identified from the reference section of the selected studies from the original search. We included articles published since inception that described the health effects or impacts of RIFA. Articles were excluded if their full texts were unavailable, if they studied non-human subjects or evaluated the health effects of other ant species concurrently that could not be disaggregated.

### 2.2. Data Analysis

For this analysis we followed the recommendations of Green et al. for a narrative overview model [13] to form a broad narrative synthesis of formerly published studies. The full text of the selected studies and other data were examined and information relevant to the review was extracted. Sting rates, RIFA sensitisation and anaphylaxis were divided into groups by study characteristics and were reported to articulate broader similarities and differences among and between the groups.

## 3. Prevalence of RIFA Stings

The majority of the relevant published literature and evidence on the human health impacts of RIFA comes from the USA, Taiwan and China, where RIFA have become established. There is significant variation in the estimates of the human burden of RIFA stings each year in infested areas (Table 1). Over half the population in infested areas is exposed to a sting at least once in their first 20 years of life and virtually the whole adult population has been stung at least once [14].

In heavily infested areas, sting attack rates range from 8.5 to 51% of the population per year. There is a significant variation in the reported rates of RIFA stings in the United States, with some estimates suggesting that 50–89% of people residing in an infested area will be stung each year [9,23], whereas other more conservative estimates put the figures at between 30 and 60% [24]. In 2018, more than 60,000 hectares of land were occupied by RIFA in Taiwan [25], and of the 10,127 Taiwanese residents who encountered RIFA, 3819 were stung by RIFA (equates to an annual sting rate of 37.71%) [22]. Additionally, RIFA were also detected in over 390 counties of 15 provinces in China [25]. Different studies conducted in China reported annual sting rates of 8.5% [19], 27.8% [20], and 30% [12] of residents in RIFA-infested areas.

The variation in rates of RIFA sting may be related to the extent of RIFA infestation, population behavior (degree of outdoor work and potential exposure), and environmental conditions impacting RIFA behavior. In overview, an annual RIFA attack rate of around 30% is a reasonable estimate [26,27].

## 4. Allergic Sensitisation

The protein component in RIFA venom includes four known allergens, designated as Sol i 1 to 4 [28], that can induce allergic sensitisation (indicated by the presence of RIFA-specific immunoglobulin E) in exposed individuals. Sensitisation to RIFA venom was detected in 38.3% of children residing in a RIFA-infested area in the United States, and varied according to their age: 35.7% in children between 2 and 5 years old and 57.5% in children between 11 and 20 years old [18]. Similarly, random screening of blood from adults in Georgia, USA, performed in 2003 found RIFA-specific immunoglobulin E (IgE) in 17% of those sampled [29]. In 2021, researchers evaluated sera from 106 participants from a RIFA-infested area in Maryland, USA, and reported that the RIFA allergic sensitisation rate in the area ranged from 19.1% to 24.1% [30]. Other researchers evaluated 703 patients in a RIFA-infested area, who were referred to an allergy clinic for any insect venom allergy and found that RIFA venom sensitisation was present in 42% of the patients. Note that it is likely that cross-sensitisation to bees and wasps may have increased this ‘any insect venom allergy’ rate [31]. Most of the allergic sensitisation, expressed serologically to RIFA venom, is clinically not relevant as no allergic reaction develops to subsequent ant stings [32]. Based on the data, it is reasonable to expect that approximately 25% of those who are stung by RIFA will develop allergic sensitisation (i.e., IgE) that is concordant with the lower end of the Hymenoptera venom sensitisation in the general population [33].

## 5. Clinical Reactions to RIFA Sting

A RIFA sting can cause a range of clinical reactions. RIFA venom is composed of approximately 5% water-soluble components, of which 0.1% (*w/w*) is protein allergens collectively known as Sol i and 95% is water-insoluble alkaloids, predominantly piperidines, known as solenopsins [6,34,35]. Reactions to RIFA venom include: (a) pseudo pustule, a small and localised reaction to the piperidine alkaloids; (b) large local reactions larger than 10 cm in diameter and associated with localised erythema (redness) and pruritus (itchiness); and (c) systemic reactions including anaphylaxis, which is a severe life-threatening allergic response that can be fatal [9].

## 6. Pseudo Pustule

**Figure 2:**
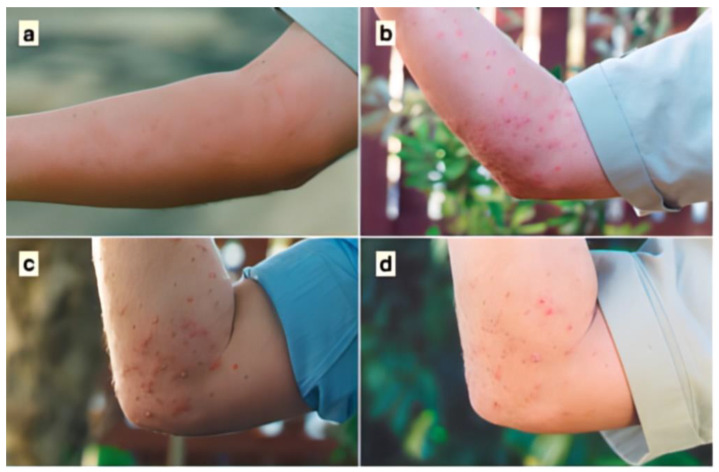
Pseudo pustule clinical progression of red imported fire ant stings (hallmark sign). (**a**) Five minutes after the event, showing raised welts at sting sites; (**b**) 18 h after, showing typical pustules; (**c**) 48 h after; and (**d**) seven days after the event (Solley et al. 2002) [37].

The most frequent reaction to an undisturbed (non-scratched) RIFA sting is a sterile 1–2 mm pseudo pustule (blister) on the skin caused by the piperidine alkaloids in RIFA’s venom [10,36], as can be seen in Figure 2 [37]. The presence of these blisters, with an appropriate history of a painful ant sting, is the hallmark sign of a RIFA sting [9]. A severe burning sensation is the immediate response to the venom [10]. After a few minutes, the sensation stops, and within two hours, a raised, red swelling appears [38]. Within four hours, blisters form in the sting location. This reaction is not an allergic reaction [38].

## 7. Large Local Reactions

Large local reactions may appear at the sting site in RIFA-venom-sensitised individuals [39,40]. This reaction occurs at the same time as the emergence of the pseudo pustule. The large local reactions are probably IgE-mediated late-phase cutaneous inflammatory reactions [41]. The reaction is characterised by extreme itchiness and the development of a large, raised, red welt at the sting site [40]. The swelling and severe itching increase over the course of 6 to 12 h and develop into a sizeable area of uncomfortable swelling. Within 24 to 48 h, these reactions reach their peak size and resolve over 7–10 days. Significant swelling and damage to the underlying blood vessels can affect the extremities [40].

In the literature, there is limited information regarding the annual rate of large local reactions due to RIFA stings. A study reported that large local reactions can occur in up to 56% of the individuals stung by RIFA [42]. A recent study from Taiwan reported that out of 3819 residents who were stung by RIFA, 802 (21.0%) had a localised allergic reaction (wheal-and-flare reaction) to its stings in a year [22]. A limitation of this study was that the large allergic reaction definition was ill-defined and it may have included pseudo pustule reactions as well [22]. The rate of large local reactions for other Hymenoptera (bees, wasps, and ants) is around 2.4–26.4% [43]. Hence, the rates suggested by Yu-Sheng et al. seem to be reasonable [22].

## 8. Systemic Reactions

Systemic allergic reactions to a RIFA sting can range from skin manifestations (generalised urticaria or angioedema) to life-threatening anaphylaxis [44]. The World Allergy Organisation (WAO) defines anaphylaxis as “a serious systemic hypersensitivity reaction that is usually rapid in onset and may cause death” [45]. Severe anaphylaxis is distinguished by a potentially life-threatening compromise in respiratory and/or circulatory functions, and it can manifest without the typical skin features or the presence of circulatory shock [45].

One RIFA sting can be enough to cause a potentially fatal anaphylaxis reaction [10]. This reaction usually develops within minutes of the sting, but in rare cases may develop up to 2 h after the sting [9]. Adults are more prone than children to suffer from severe systemic allergic reactions [46]. This is probably due to the fact that as people get older, they are more likely to have underlying health conditions, and are more likely to have developed sensitisation to RIFA venom following multiple prior stings [47]. For these reasons, older patients experience more severe reactions overall than younger ones [46]. Moreover, men are more likely than women to experience anaphylaxis due to RIFA stings, which may be due to behavioural differences leading to a greater exposure to RIFA [48]. In addition, reactions to RIFA stings vary with the season and number of stings, with a greater rate of systemic reactions occurring in summer and following multiple stings [48,49]. Also, a large number of simultaneous stings, also known as a RIFA mass attack, may prime an individual for a severe allergic reaction to “a subsequent single sting” and then be followed by a single-sting anaphylaxis [50]. A major factor related to developing an allergic response to a RIFA sting appears to be the frequency of stings; a relatively brief sting interval or several stings in succession might increase the risk of a systemic allergic reaction, shifting the disease’s natural course from asymptomatic sensitisation to RIFA venom allergy [14]. The aggressive nature of RIFA and its higher frequency of stings may explain why RIFA stings induce allergic reactions more often than bees or wasps [14].

The epidemiological estimates of the burden from anaphylaxis due to RIFA stings vary in the literature. A survey completed in 1989 by 2022 physicians who treated 20,755 patients for RIFA stings estimated that 413 patients (2%) had been treated for life-threatening anaphylaxis [26]. Taber suggested a more conservative estimate of 0.5% of individuals stung by RIFA will suffer an anaphylaxis [51]. In a study published in 1977, it was estimated that nearly 4 per 100,000 population (0.004%) in the southeastern United States developed new systemic allergic reactions to RIFA stings per year [52]. A recent study from Taiwan reported that from 3819 residents who were stung by RIFA, 106 individuals (2.78%) had an anaphylactic reaction to a RIFA sting [22]. Despite these relatively high rates of anaphylaxis, reports of fatalities due to RIFA-induced anaphylaxis are rare [9,11,12,53]. Thus, based on these data, it is estimated that between 0.5 and 2.0% of those stung by RIFA will develop an anaphylactic reaction in a given year.

## 9. Rate of Health Services Usage Due to RIFA

The use of health services due to encounters with RIFA can be significant in infested areas, making it a public health concern within both urban and rural communities. In Georgia, USA, approximately 5% of those stung by RIFA required physician management [54]. A survey of physicians in South Carolina, USA, estimated that more than 33,000 (94 per 10,000 population, or 0.94%) sought medical attention for RIFA stings, and of these, 660 people (1.9 per 10,000 population, or 0.02%) were treated for anaphylaxis [55]. In the southeastern United States, it was estimated that more than 200,000 people (around 1.5% of those stung) required medical treatment in 1995 [27]. Of the 3819 Taiwanese stung by RIFA, 288 (7.54%) sought medical treatment, and of those, 70 people (24.3%) sought medical treatment because they had an anaphylactic reaction to the sting [22]. The majority of those who seek medical attention do so for non-systemic reactions, but up to 16% are treated for generalised allergic reactions [56]. Based on these data, it is estimated that between 0.94% and 7.5% of those stung will attend health services.

## 10. Impact on Biodiversity and Ecosystem Health

The loss of biodiversity and detrimental impacts of RIFA on biodiversity and ecosystem functioning has been well documented [7]. These ants play a significant role in the loss of native species and the services and functions they provide in the ecosystem [57]. Of particular note, RIFA are highly effective at attacking and killing native arthropods, interfering with the flower-visiting behaviour of insects, that then impacts the reproductive capabilities of plants [7]. Further, mutualisms between RIFA and honeydew-producing hemipterans were reported previously; this can lead to a rapid population increase in these pests and their harmful effects on plants and crops [58]. Seeds of plants serve as crucial nutritional sources for RIFA, capable of causing harm to newly sown seeds before their germination [59]. The soil at nesting sites may be altered, further disrupting farming [7,60]. RIFA have decreased the diversity and abundance of native ant species that function as biological control of other species, affecting the local ecosystem balance [7,61]. Seabirds and mammals that occupy burrows are likely susceptible to RIFA depredation of their young [4] and are affected indirectly by the reduction in invertebrate densities (food source) that occurs in heavily RIFA-infested areas. These negative effects on animal health can have a cascading effect on ecosystem health by reducing the numbers of native predators that may introduce imbalances. Hence, it is possible that species that determine pathologies in humans become more common [3]. As such, RIFA likely have other impacts on human well-being and food security. Indeed, a recent Australian review concluded that there would be multiple impacts from an uncontrolled RIFA spread, including loss of 10% of agriculture cropping as well as a 20% reduction in livestock output significantly contributing to an estimated AU$ 2 billion in economic losses [62].

The animal and ecosystem health impacts are likely to be exacerbated in the fragile ecology found in the Pacific Islands. There can be other adverse effects on human health besides stinging attacks, for example, the potential increase of a vector species (i.e., mosquito species) of tropical diseases due to a reduction in its natural predators (i.e., spider species) due to RIFA predation [7]. There are also food security concerns that could be attributed to RIFA, for example, damage to seedlings and crops due to RIFA predation and mutualisms with pests [7]. Moreover, their effect on the flower-visiting behaviour of insects could have a significant impact on reducing crop yield [7]. In this context, there is insufficient acknowledgment of the necessity to establish connections among sectors responsible for human, animal, and plant health. This leads to an approach to biosecurity and surveillance that is both fragmented and inefficient.

## 11. Projections of Potential Health Impacts in Australia

RIFA have a concerning presence in Australia, with their invasive spread causing alarm in various regions. RIFA were first detected in Australia at two sites in Brisbane, Queensland, in 2001 [37,63]. A fire ant eradication program, in conjunction with robust community engagement, demonstrated a proactive approach that potentially averted economic losses in Australia [64]. Unfortunately, eradication efforts have faced challenges and RIFA continue to spread. In 2001, in southeast Queensland, 37,723 hectares were being treated. In 2017, the range of RIFA infestation had grown to 480,000 hectares and in 2021 to 750,000 hectares [62]. In November 2023, five RIFA nests were detected in northeastern NSW, the first report of RIFA crossing into New South Wales from Queensland [65].

We attempted to predict the health impacts to health services if RIFA becomes established Australia-wide. We used the international health impacts estimates described in the literature and governmental reports described above, and we followed a similar approach employed by Wylie and Janssen-May [64]. To do so, we have had to make certain assumptions, namely: (1) RIFA becomes established in almost all Australian states and territories; (2) all Australians in RIFA-habitable areas are exposed; (3) states and federal governments of Australia have adopted a ‘management’ approach to the pest, that is, there is no central, coordinated attempt at eradication or containment but quarantine zones may be employed to slow the spread as in the US; and (4) US-manufactured RIFA venom immunotherapy is not used widely (currently not TGA approved). We acknowledge that these factors, and therefore the human health impacts, may differ amongst each country and territory in the Western Pacific Region.

**Figure 3:**
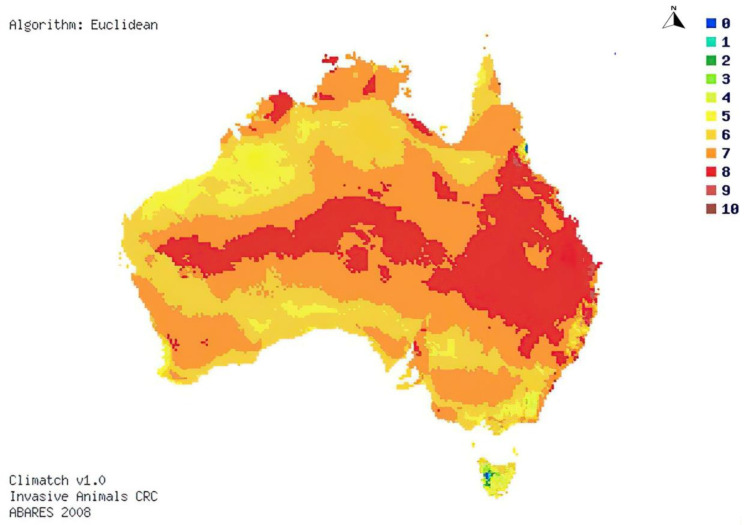
Map of potential distribution of red imported fire ants in Australia. Increasing scores represent increased habitat suitability for red imported fire ants: Scores 0–4 (below threshold, or not suitable for red imported fire ants habitat) and scores 5–10 (above threshold, or suitable for red imported fire ants habitat) [66].

A 2017 Climatch analysis was undertaken by the Australian Bureau of Agricultural and Resource Economics and Sciences (ABARES) to determine areas within Australia that would be suitable for RIFA by comparing climatic matches in Australia to that of RIFA distribution worldwide (Figure 3) [66]. This analysis determined that almost all Australians (98.5%) live in areas suitable for RIFA to thrive [66].

The population of Australia was 26,473,055 in March 2023 [67], and the RIFA-exposed Australian population would be 26,067,542 [66]. If it is assumed that one third of the population is stung by RIFA per year, this will mean that approximately 8,688,312 people would be stung in a given year. Of those stung, 2,172,077 people (a quarter of those stung) would develop allergic sensitisation, and between around 43,441 and 173,766 (from 0.5% to 2% of those stung) may develop systemic allergic reactions which would require medical attention each year. Furthermore, extrapolating from health service usage estimates from Taiwan [22], if RIFA were to become established in Australia there would be between around 78,194 and 651,623 people (from 0.94% to 7.5%) who would seek medical consultation due to RIFA stings each year, and most of the consultations would be due to local reactions [56].

To put the previous estimation into perspective, the *Myrmecia pilosula* (Jack Jumper ant) are responsible for 60% of all ant sting anaphylaxis incidents in Australia [23], between around 10 and 14% of the population exposed are stung and of those, from 2 to 3% will develop systemic allergic reactions [68]. A report by the Australian Institute of Health and Welfare in 2017 determined that *Apis mellifera* (honeybee) stings were the most common type of venomous creatures responsible for hospitalisations in Australia [69]. Approximately 6–9% of the population exposed are stung each year and of those, 0.3–2.8% will develop a systemic reaction [68,70]. In comparison, the RIFA sting rates are much higher than both Jack Jumper ants and honeybees, but their anaphylaxis rates are similar.

## 12. Ecosystem Impacts Compound Human Health Impacts

A significant number of bird species may be affected by RIFA invasion in Australia like *Alectura lathami* (Australian brush-turkey), *Turnix melanogaster* (black-breasted button-quail), and rainbow bee-eater (*Merops ornatus*) amongst others [71]. Iconic Australian mammal species could be affected as well, for example, *Ornithorhynchus anatinus* (platypus) and *Tachyglossus aculeatus* (short-beaked echidna) [71]. Moreover, invertebrate ecosystems are anticipated to undergo influences resulting from direct predation, resource competition, and interference with symbiotic relationships. The potential ramifications extend to populations of beetles, ticks, spiders, and flies, as well as land mollusks and various butterfly species [63]. These detrimental effects in animal and ecosystem health could cascade, compound, and amplify other human health impacts. For example, it could determine an increase in *Aedes notoscriptus*, the Australian backyard mosquito, population. This mosquito is likely to transmit *Mycobacteria ulcerans*, the pathogen responsible for Buruli Ulcer in southeastern Australia [72], as well as being a competent host for various viruses, from dengue to chikungunya, as well as for the filarial parasite, *Dirofilaria* immitis [73]. As yet, estimation of such complex system effects, involving feedback loops and dependencies of different durations, is barely represented in the human health literature concerning RIFA. Nevertheless, such One Health effects are likely significant, and worthy of future study given the pleiotropic impacts of this highly effective and adaptable invasive species.

## 13. Future Directions and Recommendations

We have identified several knowledge gaps in the existing literature. Firstly, there is a deficiency in up-to-date health services usage data related to RIFA, as the majority of evidence originates from studies conducted prior to the 2000s. Secondly, despite the acknowledged health impacts of RIFA, there exists limited evidence on the associated health economic costs. Thirdly, a clear diagnostic methodology to differentiate between asymptomatic sensitisation and clinical allergy is lacking. Additionally, a scarcity of published data on RIFA stings, allergic sensitisation, anaphylaxis, and health service usage rates are observed, particularly in areas where *S. invicta* is native in South America. Although crude estimates of health impacts have been presented in the context of RIFA potentially becoming established in Australia, there is a pressing need for robust prediction modelling in other areas of the Western Pacific Region. These models should consider factors such as potential expansion, seasonality, and individual predisposing factors.

RIFA pose a clear danger to the Western Pacific Region. Consequently, the potential for RIFA to establish itself as an invasive species will present a substantial and costly health issue for each affected country or territory. A significant challenge resides in the readiness of governments to actively confront the problem posed by RIFA [1]. Preparedness measures, such as early detection surveillance systems for RIFA, response plans, and the necessary infrastructure, may incur substantial costs. On this topic, and consistent with One Health principles, the IPBES has recommended coordinated efforts and collaboration across national, international and regional frameworks [1]. Additionally, it involves the formulation of effective national implementation strategies, the fostering of shared efforts, commitments, and a clear understanding of the specific roles assumed by all stakeholders. Further, addressing the issue requires enhancing policy coherence, engaging with governmental sectors, the industrial domain, scientific communities, indigenous peoples, local communities, and the broader public [1]. It also involves securing and allocating funding for innovative research and environmentally conscious technology, as well as fortifying information systems, infrastructure and the sharing of data [1].

Implementation of efficient biosecurity measures and pest management is imperative. The first barrier should be the implementation of effective detection systems at ports [4]. New Zealand and Australia employ regular port surveillance, coupled with incursion response readiness and actions if necessary [4]. The use of sea container hygiene systems to mitigate the risk associated with container contamination is also recommended [4]. In addition to awareness campaigns and surveillance, successful eradication programs to identify and eradicate new RIFA incursions would avoid substantial health impacts and related costs. In the event of a continued spread of RIFA through the region, public health awareness needs to be raised, and health systems need to be supported to manage the increased usage and hospitalisation.

## 14. Conclusions

We found that the impacts of RIFA on human health are considerable in infested areas. Approximately one-third of the population residing in RIFA-infested areas experiences one or more stings each year [9]. Around 25% of those stung will develop allergic sensitisation, characterised by elevated venom-specific IgE levels in response to RIFA venom [51]. The allergic reactions to RIFA stings include large local reactions (20% of those stung) and systemic reactions (0.5–2.0% of those stung). Local biodiversity is disrupted by invading RIFA, which can lead to other adverse effects on human health and food security concerns. The Western Pacific Region has notable heterogeneity, arising from cultural diversity, diverse developmental stages, and environmental distinctions [4]. Nevertheless, many areas within the Pacific Region, like the Pacific Island Countries and Territories, have lifestyles oriented towards outdoor activities, often have more restricted access to emergency healthcare services, and have high rates of manual agricultural practices [4]. The potential RIFA health impacts are likely to be higher in these areas. Due to global shipping and trading and the lack of an effective surveillance and early detection system, there is a high and ongoing threat of RIFA invasion for all countries and territories in the Western Pacific Region.

## Figures and Tables

**Table 1:** Prevalence of red imported fire ant stings in infested areas reported in the literature.

Study	Year	Region	Participants	Sting Prevalence
Clemmer and Serfling [15]	1975	New Orleans, USA	240 households	29% over 12 months
Adams and Lofgren [16]	1981	Georgia, USA	272 participants	35% over 12 months
Tracy et al. [17]	1995	Texas, USA	107 military medical students	51% over 3 weeks
Partridge et al. [18]	2008	Georgia, USA	183 participants	38.6% over 1 month
Xu et al. [19]	2006	South China	-	8.5% over 12 months
Wu et al. [20]	2005	South China	308 families	27.8% over 12 months
Zhang et al. [21]	2006	South China	59 families	32.4% over 12 months
Liu et al. [22]	2023	Taiwan	10,127 participants	37.7% over 12 months

## Data Availability

No new data were created or analysed in this study. Data sharing is not applicable to this article.

## References

[1] Roy H.E., Pauchard A., Stoett P., Renard Truong T., Bacher S., Galil B.S., Hulme P.E., Ikeda T., Sankaran K.V., McGeoch M.A. (2023). Summary for Policymakers of the Thematic Assessment Report on Invasive Alien Species and Their Control of the Intergovernmental Science-Policy Platform on Biodiversity and Ecosystem Services.

[2] Ascunce M.S., Yang C.-C., Oakey J., Calcaterra L., Wu W.-J., Shih C.-J., Goudet J., Ross K.G., Shoemaker D. (2011). Global invasion history of the fire ant Solenopsis invicta. Science.

[3] Zinsstag J., Schelling E., Waltner-Toews D., Tanner M. (2011). From “one medicine” to “one health” and systemic approaches to health and well-being. Prev. Vet. Med..

[4] Gruber M.A., Janssen-May S., Santoro D., Cooling M., Wylie R. (2021). Predicting socio-economic and biodiversity impacts of invasive species: Red Imported Fire Ant in the developing western Pacific. Ecol. Manag. Restor..

[5] Stoett P., Roy H.E., Pauchard A. (2019). Invasive alien species and planetary and global health policy. Lancet Planet. Health.

[6] Stafford C.T. (1996). Hypersensitivity to fire ant venom. Ann. Allergy Asthma Immunol..

[7] Wang L., Xu Y.-J., Zeng L., Lu Y.-Y. (2019). Impact of the red imported fire ant Solenopsis invicta Buren on biodiversity in South China: A review. J. Integr. Agric..

[8] Sánchez-Peña S.R., Patrock R.J., Gilbert L.A. (2005). The red imported fire ant is now in Mexico: Documentation of its wide distribution along the Texas-Mexico border. Entomol. News.

[9] de Shazo R.D., Williams D., Goddard J., Rockhold R., Kemp S. (2023). Stings of Imported Fire Ants: Clinical Manifestations, Diagnosis, and Treatment.

[10] Kruse B., Anderson J., Simon L.V. (2022). Fire Ant Bites.

[11] Rupp M.R., Deshazo R.D. (2006). Indoor fire ant sting attacks: A risk for frail elders. Am. J. Med. Sci..

[12] Xu Y., Huang J., Zhou A., Zeng L. (2012). Prevalence of Solenopsis invicta (Hymenoptera: Formicidae) venom allergic reactions in mainland China. Fla. Entomol..

[13] Green B.N., Johnson C.D., Adams A. (2006). Writing narrative literature reviews for peer-reviewed journals: Secrets of the trade. J. Chiropr. Med..

[14] Antonicelli L., Bilò M.B., Bonifazi F. (2002). Epidemiology of Hymenoptera allergy. Curr. Opin. Allergy Clin. Immunol..

[15] Clemmer D.I., Serfling R.E. (1975). The imported fire ant: Dimensions of the urban problem. South. Med. J..

[16] Adams C., Lofgren C. (1981). Red imported fire ants (Hymenoptera: Formicidae): Frequency of sting attacks on residents of Sumter County, Georgia. J. Med. Entomol..

[17] Tracy J.M., Demain J.G., Quinn J.M., Hoffman D.R., Goetz D.W., Freeman T.M. (1995). The natural history of exposure to the imported fire ant (*Solenopsis invicta*). J. Allergy Clin. Immunol..

[18] Partridge M.E., Blackwood W., Hamilton R.G., Ford J., Young P., Ownby D.R. (2008). Prevalence of allergic sensitization to imported fire ants in children living in an endemic region of the southeastern United States. Ann. Allergy Asthma Immunol..

[19] Xu Y., Lu Y.Y., Zeng L., Xi Y.B., Huang J. (2006). Study on location expansion of red imported fire ant Solenopsis invicta Buren. J. South China Agric. Univ..

[20] Nengjian W., Wencheng L., Huiming L., Zidian H., Jianfeng H., Kangbin L., Chun Y., Jianyi K., Kangshou X. (2005). A survey on human bitten by red imported fire ants in mainland for the first time. Chin. J. Vector Biol. Control.

[21] Zhang Q., Lin L., Chen H., Chen P., Lu W., Li Y. (2006). An investigation on the first human death incident caused by the bite of red imported fire ants. Dis. Surveill..

[22] Liu Y.-S., Huang S.-A., Lin I.-L., Lin C.-C., Lai H.-K., Yang C.-H., Huang R.-N. (2021). Establishment and social impacts of the red imported fire ant, Solenopsis invicta,(Hymenoptera: Formicidae) in Taiwan. Int. J. Environ. Res. Public Health.

[23] Wanandy T., Mulcahy E., Lau W.Y., Brown S.G.A., Wiese M.D. (2022). Global View on Ant Venom Allergy: From Allergenic Components to Clinical Management. Clin. Rev. Allergy Immunol..

[24] Pereira R.M., Williams D.F., Davis T.S., Oi D.H., Bolton H.T., Horton P.M., Williams H.G. Imported fire ants and their management. Proceedings of the 1st Quail Management Short Course.

[25] Wang L., Zeng L., Xu Y., Lu Y. (2020). Prevalence and management of Solenopsis invicta in China. NeoBiota.

[26] Stafford C., Hutto L., Rhoades R., Thompson W., Impson L. (1989). Imported fire ant as a health hazard. South. Med. J..

[27] Hoffman D. (1995). Fire ant venom allergy. Allergy.

[28] Hoffman D.R. (1993). Allergens in Hymenoptera venom XXIV: The amino acid sequences of imported fire ant venom allergens Sol i II, Sol i III, and Sol i IV. J. Allergy Clin. Immunol..

[29] Caplan E.L., Ford J.L., Young P.F., Ownby D.R. (2003). Fire ants represent an important risk for anaphylaxis among residents of an endemic region. J. Allergy Clin. Immunol..

[30] Park H.J., Brooks D.I., Chavarria C.S., Wu R.L., Mikita C.P., Beakes D.E. (2022). Combining discordant serum IgE and skin testing improves diagnostic and therapeutic accuracy for Hymenoptera venom hypersensitivity immunotherapy. J. Allergy Clin. Immunol. Pract..

[31] Freeman T.M. (1997). Hymenoptera hypersensitivity in an imported fire ant endemic area. Ann. Allergy Asthma Immunol..

[32] Perčič S., Bojanić L., Košnik M., Kukec A. (2022). Natural History of the Hymenoptera Venom Sensitivity Reactions in Adults: Study Design. Int. J. Environ. Res. Public Health.

[33] Schäfer T., Przybilla B. (1996). IgE antibodies to Hymenoptera venoms in the serum are common in the general population and are related to indications of atopy. Allergy.

[34] Chen L., Fadamiro H.Y. (2009). Re-investigation of venom chemistry of Solenopsis fire ants. I. Identification of novel alkaloids in S. richteri. Toxicon.

[35] Fox E.G.P., Solis D.R., Dos Santos L.D., dos Santos Pinto J.R.A., da Silva Menegasso A.R., Silva R.C.M.C., Palma M.S., Bueno O.C., de Alcântara Machado E. (2013). A simple, rapid method for the extraction of whole fire ant venom (Insecta: Formicidae: Solenopsis). Toxicon.

[36] Jung R., Derbes V., Burch A. (1963). Skin Response to Solenamine, a Hemolytic Component of Fire-ant Venom. Int. J. Dermatol..

[37] Solley G.O., Vanderwoude C., Knight G.K. (2002). Anaphylaxis due to red imported fire ant sting. Med. J. Aust..

[38] Goddard J., Jarratt J., de Castro F.R. (2000). Evolution of the fire ant lesion. JAMA.

[39] deShazo R.D., Butcher B.T., Banks W. (1990). Reactions to the stings of the imported fire ant. N. Engl. J. Med..

[40] deShazo R.D., Quinn J.M. (2023). Patient Education: Imported Fire Ants (Beyond the Basics).

[41] Tripolt P., Arzt-Gradwohl L., Čerpes U., Laipold K., Binder B., Sturm G.J. (2020). Large local reactions and systemic reactions to insect stings: Similarities and differences. PLoS ONE.

[42] deShazo R.D., Griffing C., Kwan T.H., Banks W., Dvorak H. (1984). Dermal hypersensitivity reactions to imported fire ants. J. Allergy Clin. Immunol..

[43] Bilo B., Rueff F., Mosbech H., Bonifazi F., Oude-Elberink J.N.G., EAACI Interest Group on Insect Venom Hypersensitivity (2005). Diagnosis of Hymenoptera venom allergy. Allergy.

[44] Reber L.L., Hernandez J.D., Galli S.J. (2017). The pathophysiology of anaphylaxis. J. Allergy Clin. Immunol..

[45] Turner P.J., Worm M., Ansotegui I.J., El-Gamal Y., Rivas M.F., Fineman S., Geller M., Gonzalez-Estrada A., Greenberger P.A., Tanno L.K. (2019). Time to revisit the definition and clinical criteria for anaphylaxis?. World Allergy Organ. J..

[46] Golden D.B. (2007). Insect sting anaphylaxis. Immunollogy Allergy Clin. N. Am..

[47] Vega A., Castro L. (2019). Impact of climate change on insect–human interactions. Curr. Opin. Allergy Clin. Immunol..

[48] Braun C.T., Mikula M., Ricklin M.E., Exadaktylos A.K., Helbling A. (2016). Climate data, localisation of the sting, grade of anaphylaxis and therapy of Hymenoptera stings. Swiss Med. Wkly..

[49] Auerswald L., Lopata A. (2005). Insects—Diversity and allergy: Review article. Curr. Allergy Clin. Immunol..

[50] Pucci S., Antonicelli L., Bilo M., Garritani M., Bonifazi F. (1994). Shortness of interval between two stings as risk factor for developing Hymenoptera venom allergy. Allergy.

[51] Taber S.W. (2000). Fire Ants.

[52] Rhoades R., Schafer W., Newman M., Lockey R., Dozier R., Wubbena P., Townes A., Schmid W., Neder G., Brill T. (1977). Hypersensitivity to the imported fire ant in Florida. Report of 104 cases. J. Fla. Med. Assoc..

[53] Rhoades R.B., Stafford C.T., James F.K. (1989). Survey of fatal anaphylactic reactions to imported fire ant stings. J. Allergy Clin. Immunol..

[54] Yeager W. (1978). Frequency of fire ant stinging in Lowndes County, Georgia. J. Med. Assoc. Ga..

[55] Caldwell S., Schuman S.H., Simpson W. (1999). Fire ants: A continuing community health threat in South Carolina. J. South Carol. Med. Assoc. (1975).

[56] Triplett R. (1976). The imported fire ant: Health hazard or nuisance?. South. Med. J..

[57] Stuble K.L., Chick L.D., Rodriguez Cabal M.A., Lessard J.P., Sanders N.J. (2013). Fire ants are drivers of biodiversity loss: A reply to King and Tschinkel (2013). Ecol. Entomol..

[58] Wu D., Zeng L., Xu Y. (2015). Impact of Solenopsis invicta and its mutualism with aphids on flower-visiting behavior of insects on mungbean, Vigna radiata. J. Environ. Entomol..

[59] Morrison J.E., Williams D.F., Oi D.H., Potter K.N. (1997). Damage to dry crop seed by red imported fire ant (Hymenoptera: Formicidae). J. Econ. Entomol..

[60] Carlson S.R., Whitford W.G. (1991). Ant mound influence on vegetation and soils in a semiarid mountain ecosystem. Am. Midl. Nat..

[61] Porter S.D., Savignano D.A. (1990). Invasion of polygyne fire ants decimates native ants and disrupts arthropod community. Ecology.

[62] Scott-Orr H., Gruber M., Zacharin W. (2021). National Red Imported Fire Ant Eradication Program Strategic Review August 2021.

[63] Moloney S., Vanderwoude C. (2002). Red Imported Fire Ants: A threat to eastern Australia’s wildlife?. Ecol. Manag. Restor..

[64] Wylie F.R., Janssen-May S. (2017). Red imported fire ant in Australia: What if we lose the war?. Ecol. Manag. Restor..

[65] Queensland Fire Ant Infestation Marches over the NSW Border. https://www.abc.net.au/news/2023-11-25/qld-fire-ant-infestation-marches-over-the-nsw-border/103150896.

[66] Janssen S. (2017). Ten Year Eradication Plan, National Red Imported Fire Ant Eradication Program, South East Queensland, 2017–2018 to 2026–2027.

[67] National, State and Territory Population. https://www.abs.gov.au/statistics/people/population/national-state-and-territory-population/mar-2023.

[68] Brown S.G., Franks R.W., Baldo B.A., Heddle R.J. (2003). Prevalence, severity, and natural history of jack jumper ant venom allergy in Tasmania. J. Allergy Clin. Immunol..

[69] AIHW (2021). Venomous Bites and Stings, 2017–18.

[70] Douglas R.G., Weiner J.M., Abramson M.J., O’Hehir R.E. (1998). Prevalence of severe ant-venom allergy in southeastern Australia. J. Allergy Clin. Immunol..

[71] Lach L., Barker B. (2013). Assessing the Effectiveness of Tramp Ant Projects to Reduce Impacts on Biodiversity. A Report Prepared for the Australian Government Department of Sustainability, Environment, Water, Population, and Communities.

[72] Mee P.T., Buultjens A.H., Oliver J., Brown K., Crowder J.C., Porter J.L., Hobbs E.C., Judd L.M., Taiaroa G., Puttharak N. (2023). A transmission chain linking Mycobacterium ulcerans with Aedes notoscriptus mosquitoes, possums and human Buruli ulcer cases in southeastern Australia. bioRxiv.

[73] Trewin B.J., Pagendam D.E., Zalucki M.P., Darbro J.M., Devine G.J., Jansen C.C., Schellhorn N.A. (2019). Urban Landscape Features Influence the Movement and Distribution of the Australian Container-Inhabiting Mosquito Vectors Aedes aegypti (Diptera: Culicidae) and Aedes notoscriptus (Diptera: Culicidae). J. Med. Entomol..

